# Supplementary material for: The role of prehabilitation in improving brain health and cognition after chemotherapy in patients with colorectal cancer: Study protocol of the Chemo Brain Prehab Project
Source: PLoS One. 2026 Mar 3;21(3):e0341996. doi: 10.1371/journal.pone.0341996 (PMC12956118; doi:10.1371/journal.pone.0341996)
Supplement: S1 File — (DOCX) [file pone.0341996.s001.docx]

Title: **Improving Brain Health After Chemotherapy Through Prehabilitation**

Short Title**: ChemoBrain Prehab Project**

**RESEARCH REFERENCE NUMBER:**

**STUDY REGISTRY NUMBER AND DATE:**

**PROTOCOL VERSION NUMBER AND DATE:** Version 1.0 (04/12/2025)

**SPONSOR NUMBER:**

**FUNDER NUMBER:**

## SIGNATURE PAGE

The undersigned confirm that the following protocol has been agreed and accepted and that the Chief Investigator agrees to conduct the study in compliance with the approved protocol and will adhere to the national guidelines for the conduct of clinical research.

I agree to ensure that the confidential information contained in this document will not be used for any other purpose other than the evaluation or conduct of the clinical investigation without the prior written consent of the Sponsor

I also confirm that I will make the findings of the study publicly available through publication or other dissemination tools without any unnecessary delay and that an honest accurate and transparent account of the study will be given; and that any discrepancies and serious breaches of Good Clinical Practice (GCP) from the study as planned in this protocol will be explained.

| **For and on behalf of the Study Sponsor:** | | |
| --- | --- | --- |
| Signature:  ...................................................................................................... |  | Date: ....../....../...... |
| Name (please print):  ...................................................................................................... |  |  |
| Position: ...................................................................................................... |  |  |
| **Chief Investigator:** | | |
| Signature: ...................................................................................................... |  | Date:  ....../....../...... |
| Name: (please print):  ...................................................................................................... |  |  |

Contents

[Title: 1](#_Toc208519886)

[Short Title 1](#_Toc208519887)

[SIGNATURE PAGE 2](#_Toc208519888)

[i. KEY STUDY CONTACTS 5](#_Toc208519889)

[ii. LIST OF ABBREVIATIONS 7](#_Toc208519890)

[iii. STUDY SUMMARY 8](#_Toc208519891)

[iv. FUNDING 10](#_Toc208519892)

[v. ROLE OF STUDY SPONSOR AND FUNDER 10](#_Toc208519893)

[vi. ROLES AND RESPONSIBILITIES OF STUDY MANAGEMENT COMMITEES/GROUPS & INDIVIDUALS 10](#_Toc208519894)

[vii. PROTOCOL CONTRIBUTORS 11](#_Toc208519895)

[viii. KEY WORDS: 12](#_Toc208519896)

[ix. STUDY FLOW CHART 13](#_Toc208519897)

[1. BACKGROUND & RATIONALE 14](#_Toc208519898)

[2. ASSESSMENT AND MANAGEMENT OF RISK 14](#_Toc208519899)

[3. OBJECTIVES AND OUTCOME MEASURES/ENDPOINTS 16](#_Toc208519900)

[3.1. Primary objective 16](#_Toc208519901)

[3.2. Secondary objectives 16](#_Toc208519902)

[3.3. Outcome measures/endpoints 16](#_Toc208519903)

[3.4. Primary endpoint/outcome 17](#_Toc208519904)

[3.5. Secondary endpoint/outcome 17](#_Toc208519905)

[3.6. Table of endpoints/outcomes 17](#_Toc208519906)

[4. STUDY DESIGN 18](#_Toc208519907)

[5. STUDY SETTING 18](#_Toc208519908)

[6. PARTICIPANT ELIGIBILITY CRITERIA 19](#_Toc208519909)

[6.1. Inclusion criteria 19](#_Toc208519910)

[6.2. Exclusion criteria 19](#_Toc208519911)

[7. STUDY PROCEDURES 19](#_Toc208519912)

[7.1. Recruitment 19](#_Toc208519913)

[7.2. Consent 20](#_Toc208519914)

[7.3. The randomisation scheme 21](#_Toc208519915)

[7.4. Baseline data 21](#_Toc208519916)

[7.5. Study Intervention and Assessments 21](#_Toc208519917)

[7.6. Withdrawal criteria 28](#_Toc208519918)

[7.7. Storage and analysis of clinical samples 28](#_Toc208519919)

[7.8. Definition of the end of Study 29](#_Toc208519920)

[8. SAFETY REPORTING 29](#_Toc208519921)

[8.1. Definitions 29](#_Toc208519922)

[8.2. Workflow of reporting concerns to the direct care team and documentation 30](#_Toc208519923)

[9. STATISTICS AND DATA ANALYSIS 31](#_Toc208519924)

[9.1. Sample size calculation 31](#_Toc208519925)

[9.2. Planned recruitment rate 31](#_Toc208519926)

[9.3. Statistical analysis plan 31](#_Toc208519927)

[9.4. Procedure(s) to account for missing or spurious data 32](#_Toc208519928)

[10. DATA MANAGEMENT 32](#_Toc208519929)

[10.1. Data collection tools and source document identification 32](#_Toc208519930)

[10.2. Data handling and record keeping 33](#_Toc208519931)

[10.3. Access to Data 33](#_Toc208519932)

[11. ETHICAL AND REGULATORY CONSIDERATIONS 33](#_Toc208519933)

[11.1. Research Ethics Committee (REC) review & reports 33](#_Toc208519934)

[11.2. Public and Patient Involvement (PPI) 34](#_Toc208519935)

[11.3. Regulatory Compliance 34](#_Toc208519936)

[11.4. Protocol compliance 35](#_Toc208519937)

[11.5. Data protection and patient confidentiality 35](#_Toc208519938)

[11.6. Financial and other competing interests for the Chief investigator, Principal Investigators at each site and committee members for the overall study management 36](#_Toc208519939)

[11.7. Amendments 36](#_Toc208519940)

[11.8. Post Study Care 36](#_Toc208519941)

[11.9. Access To The Final Study Dataset 36](#_Toc208519942)

[12. DISSEMINIATION POLICY 36](#_Toc208519943)

[12.1. Dissemination policy 36](#_Toc208519944)

[12.2. Authorship eligibility guidelines and any intended use of professional writers 37](#_Toc208519945)

[**13. REFERENCES** 38](#_Toc208519946)

[14. APPENDICES 39](#_Toc208519947)

## i. KEY STUDY CONTACTS

| Lead Investigator | Ms. Katie Hoad  Lancaster Medical School  Faculty of Health & Medicine  Lancaster University  Lancaster, LA1 4YG  +44 (0) 7832158964  k.hoad@lancaster.ac.uk |
| --- | --- |
| Chief Investigator | Dr Christopher Gaffney  Lancaster Medical School  Faculty of Health & Medicine  Lancaster University  Lancaster, LA1 4YG  +44 (0) 1524 593 602  [c.gaffney@lancaster.ac.uk](mailto:c.gaffney@lancaster.ac.uk) |
| Co-Investigator | Dr. Helen Nuttall  Fylde College,  Lancaster University  Lancaster, LA1 4YG  +44 (0) 1524 592 842  h.nuttall1@lancaster.ac.uk |
| Principal Investigators For Each Participating Site | Mr. Daren Subar  Department of General Surgery  East Lancashire Teaching Hospitals NHS Trust  Blackburn, BB2 3HH  +44 (0) 1524 735 604  daren.subar@elht.nhs.uk |
|  | Dr. Deborah Williamson  Department of Oncology  Lancashire Teaching Hospitals NHS Trust  Preston, PR2 9HT  +44 (0) 1772 522 984  deborah.williamson@lthtr.nhs.uk |
|  | Dr. Chan Ton  Department of Oncology  University Hospitals of Morecambe Bay NHS Foundation Trust  Royal Lancaster Infirmary  Lancaster, LA1 1BF  +44 (0) 1539 732 288  chan.ton@mbht.nhs.uk |
| Sponsor | Lancaster University,  Bailrigg, LA1 4YW  +44(0) 1524 593 017  [sponsorship@lancaster.ac.uk](mailto:sponsorship@lancaster.ac.uk) |
| Funder(s) | North West Cancer Research  Mr Alastair Richards  131 Mount Pleasant,  Liverpool, L3 5TF  alastair@nwcr.org |

## ii. LIST OF ABBREVIATIONS

AE Adverse Event

AR Adverse Reaction

BDNF Brain-Derived Neurotrophic Factor

CPET Cardiopulmonary Exercise Test

ECG electrocardiogram

EEG Electroencephalogram

ERP Event Related Potentials

GCP Good Clinical Practice

GP General Practitioner

IPAQ International Physical Activity Questionnaire

ISRCTN International Standard Randomised Controlled Study’s Number

NHS National Health Service

PAR-Q+ Physical Activity Readiness Questionnaire

PIS Participant Information Sheet

PPI Public and Patient Involvement

RCT Randomised Control Trial

REC Research Ethics Committee

SAE Serious Adverse Event

SAR Serious Adverse Reaction

SDV Source Data Verification

SOP Standard Operating Procedure

SSC Study Steering Committee

VEGF Vascular Endothelial Growth Factor

## iii. STUDY SUMMARY

| Study Title | Improving Brain Health After Chemotherapy Through Prehabilitation | |
| --- | --- | --- |
| Short Title | ChemoBrain Prehab Project | |
| Study Design | Multi-Centre Randomised Control Trial  Intervention: Prehabilitation Group  Control Group: Standard Care Group | |
| Study Participants | Stage II and III colorectal cancer patients who are undergoing chemotherapy | |
| Planned Sample Size | 86 patients with colorectal cancer were divided into Group 1: Intervention (Prehabilitation; n = 43), and Group 2: Control (Standard Care With No Prehabilitation; n = 43). | |
| Randomisation | Block randomisation will be performed at an independent site (Lancaster University), and the sequence will be shared with the recruitment team via sealed envelopes. | |
| Allocation Concealment | The study arm allocation will be provided in sealed envelopes. | |
| Counties of Recruitment | Lancashire | |
| Intervention | Supervised moderate-intensity exercise programme for participants to attend twice per week [online] and two unsupervised, self-paced exercise sessions. Totally to 4 exercise sessions per week at home. This will include warm-up and cool-down sessions. Strength training will focus on muscle groups and avoiding core exercise, which can increase the risk of excessive abdominal pressure. Grades of resistance band (or equivalent) will be provided, and regression and progression to exercise will be offered to individualise exercise programmes according to participants’ mobility and capabilities.  Participants will be provided with four-week supply of multivitamins (Forceval) before chemotherapy, and then X amount as part of standard of care (dependent on the number of rounds of chemotherapy (4-24 weeks supply)). The multivitamins will be taken in addition to the exercise programme. | |
| Control | Standard care group. | |
| Intervention duration | The maximum duration of prehabilitation participation will last a maximum of 28 weeks, however, there may be delays to treatment due to chemotherapy-related side effects (e.g., low white blood cell count). | |
| Follow up duration | 3 months | |
| Planned Study Period | 5^th^ January 2026 to 16^th^ September 2027 | |
|  | *Objectives* | *Outcome Measures* |
| Primary | Use physiological blood-based markers to establish if prehabilitation promotes an improvement in brain health in colorectal cancer patients prior to starting chemotherapy. | Brain-Derived Neurotrophic Factor (BDNF) and Vascular Endothelial Growth Factor (VEGF). |
|  | We will use neuroscientific measures of brain function (electroencephalography (EEG)) and cognitive function at baseline and post-chemotherapy to determine if prehabilitation neuroplasticity reduces the negative effects of chemotherapy on the brain. | EEG spectral ratios and oscillation analysis that focuses on brain activity during a battery of cognitive function tests.  Cognitive outcomes (executive function, memory, attention, and processing speed; with audition) |
| Secondary | We will measure changes in cognitive-related quality of life in colorectal cancer patients at Study Visit 1 and at 3 months after chemotherapy cessation to determine whether prehabilitation had an impact. | Quality-of-life questionnaire |

## iv. FUNDING

| **FUNDER(S)**  (Names and contact details of ALL organisations providing funding and/or support in kind for this study) | **FINANCIAL AND NON FINANCIAL SUPPORT GIVEN** |
| --- | --- |
| North West Cancer Research:  Mr Alastair Richards, CEO, [alastair@nwcr.org](mailto:alastair@nwcr.org).  Funding for the research study was also provided. | £237,181.19 |

## v. ROLE OF STUDY SPONSOR AND FUNDER

Lancaster University, as the Sponsor, assumes overall responsibility for the initiation and governance of the study. This includes ensuring that the research complies with relevant ethical, legal, and regulatory standards, and that appropriate risk management, insurance, and oversight mechanisms are in place. The Sponsor, in its supervisory role, will monitor and support the project through established protocols delivered by the university’s Clinical Research Governance Team, ensuring that the study is conducted to high scientific and ethical standards, and that outcomes are appropriately reported and disseminated.

North West Cancer Research, as the Funder, is a registered charity, whose role is to provide financial support to enable the research to be conducted. While the Funder may outline strategic priorities and conditions for the use of funds, it does not assume legal or regulatory responsibility in this study. The Funder is not the Sponsor and is therefore not involved in the management, oversight, or conduct of the research. The sponsor and the research team are responsible for ensuring that the study meets ethical, legal, and governance requirements. The Funder may require progress updates, a final report, and evidence of dissemination in line with its commitment to transparency and impact.

## vi. ROLES AND RESPONSIBILITIES OF STUDY MANAGEMENT COMMITEES/GROUPS & INDIVIDUALS

The public and patient involvement (PPI) steering committee will consist of individuals with lived experience of colorectal cancer, including experience of chemotherapy-related cognitive impairments (‘brain fog,’ ‘chemo-brain’), and carers of people who have undergone chemotherapy treatment. The group will be facilitated and chaired by the lead investigator, who will ensure inclusive, well-managed discussions and support the contribution of all members in informing the design, management, and dissemination of the research.

The Study Steering Committee (SSC) will provide overall oversight of the study and ensure that it is conducted according to the highest standards of scientific and ethical integrity. The committee will include the Lead Investigator (Hoad), Chief Investigator (Gaffney), Co-Investigator (Nuttall), Clinical Lead (Subar), and three academic members with expertise in clinical trial management and oversight. In alignment with the best practice in PPI, a representative from the study’s PPI advisory group will also participate in quarterly SSC meetings to ensure that the perspectives of those with lived experience inform study governance. Management challenges may arise in the timing of recruitment and enrolment into the exercise prehabilitation, especially across multiple hospital sites. Recruitment and retention issues will be addressed in our steering group meetings and monitoring of the testing protocol.

## vii. PROTOCOL CONTRIBUTORS & ROLES AND RESPONSIBILITIES

**Hoad** is a research associate currently based at Lancaster Medical School with a background in exercise physiology. She was involved in cerebrovascular and cardiovascular health in patients with stroke and during cardiac rehabilitation. Hoad is employed for 2.5 years to conduct this project. Hoad will recruit all participants, conduct all data collection (CPET/Bloods/EEG/Cognitive Battery) during hospital visits, run the intervention (exercise sessions), manage data, and analyse all data with guidance from Gaffney and Nuttall.

**Gaffney** (Lecturer in Sports Science, Lancaster University, UK) is a physiologist based in Lancaster Medical School with expertise in skeletal muscle metabolism. His experience covers research involving patients, elite athletes, and his industrial partners including work with NASA and SpaceX. Gaffney will provide oversight of the project, support the lead investigator (Hoad) in CPET and blood panels data and prepare data for publication.

**Nuttall** (Lecturer in Cognitive Neuroscience, Lancaster University, UK) is a neuroscientist with expertise in cognitive neuroscience and sensorimotor function, using auditory brainstem responses (ABRs), frequency following responses (FFRs), electroencephalography (EEG), transcranial magnetic stimulation (TMS), and motor evoked potentials (MEPs). Nuttall will support the lead investigator (Hoad), assist with all EEG and cognitive-related data, and prepare data for publication.

**Subar** is a consultant laparoscopic and Hepato-Pancreato-Biliary surgeon with an interest in minimal access surgery. He is the lead for robotic HPB in the department, which will soon be launched. He is also the lead in research and development in the Department of General Surgery. Subar will be the clinical co-investigator/lead clinician of the project and principal investigator at East Lancashire NHS Teaching Hospitals and will review lists of patients weekly throughout the recruitment window.

**Williamson** is a consultant clinical oncologist based at the Lancashire Teaching Hospital NHS Trust. Her research has focused on cancer, specifically the optimisation of chemoradiotherapy and standardisation of clinical trial outcomes. Williamson will be the clinical co-investigator of the work, principal investigator at Lancashire Teaching Hospital NHS Trust and will review lists of patients weekly throughout the recruitment window**.**

**Ton** is a consultant medical oncologist at the Royal Lancaster Infirmary, University Hospitals Morecambe Bay Trust. Ton will be the clinical co-investigator of the work, principal investigator at University Hospitals Morecambe Bay Trust, and will review lists of patients weekly throughout the recruitment window.

| viii. KEY WORDS: | Prehabilitation, Colorectal Cancer, Chemo-Brain, Chemotherapy-Induced Cognitive Impairment, Exercise, Quality of Life. |
| --- | --- |

## ix. STUDY FLOW CHART


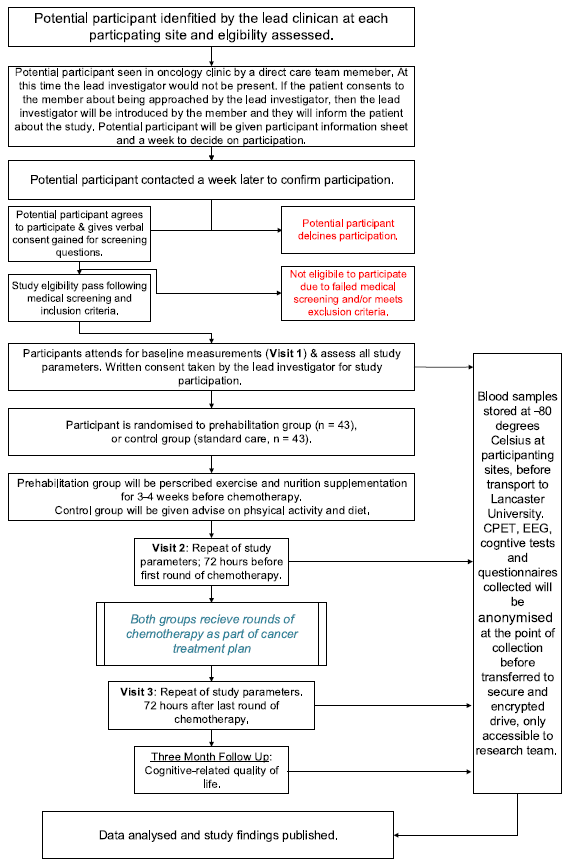


## 1. BACKGROUND & RATIONALE

Colorectal cancer is 37% higher in the North-West than the national average, and recent analysis suggests that overall survival in the North-West is the worst in England at 58.1% (95% CI: 57.3%, 58.9%)^1,2^. Colorectal cancer is treated with chemotherapy, including fluorouracil, capecitabine, and oxaliplatin. These drugs do not just kill the tumour, they also damage cells across the body, which can lead to common side effects, such as hair loss. A further common side effect is “chemo-brain,” which describes a range of symptoms, including problems with memory and thinking speed. Such symptoms reduce the quality of life and increase stress in patients.

One of the most promising new avenues for optimising patient treatment in cancer is “prehabilitation” ^3,4^ a term used to capture exercise and nutrition interventions to prepare patients before treatment. Prehabilitation has excellent surgical benefits. It can improve cardiorespiratory fitness before cancer treatment, improves functional capacity, reduces post-operative complications, and reduces hospital length of stay^3-5^, thus offering patient-centred and economic benefits. The benefits before chemotherapy are less well defined, but the mechanisms that improve outcomes for surgery remain intact in patients who undergo chemotherapy.

Exercise improves both the body and brain. For the body, it improves cardiovascular function and promotes muscle protein synthesis and skeletal muscle accretion^5^. In the brain, exercise increases growth factors in the blood, which collectively improves brain health and cognitive reserve. These include Brain-Derived Neurotrophic Factor (BDNF) and Vascular Endothelial Growth Factor (VEGF)^6^. Exercise-induced increases in new cells in the nervous system result in increased white matter volume, gray matter volume, and neural activity in the brain^7^. Exercise also promotes greater connectivity within the nervous system and the formation of new blood vessels, which increases cerebral blood flow^7^. Therefore, exercise can improve brain health and function. Exercise can help build the body up before chemotherapy breaks the body down, alleviating some side effects associated with chemotherapy. We will use an existing prehabilitation intervention we have developed, which increases fitness and reduces hospital length of stay in colorectal cancer patients undergoing surgery, to try and improve the symptoms of chemo-brain of those undergoing chemotherapy and improve quality of life. Given the known link between cardiorespiratory fitness and markers of brain health^7^, we hypothesise that prehabilitation will also improve chemo-brain symptoms resulting from chemotherapy.

## 2. ASSESSMENT AND MANAGEMENT OF RISK

**Potential Risks**

Cardiopulmonary exercise testing (CPET) carries a small but important risk of adverse events. To minimise the risk of exertional arrhythmias, cardiac events, or syncope, participants will be pre-screened using the Physical Activity Readiness questionnaire (PAR-Q+), with medical clearance obtained if needed before the first visit to the hospital for testing. Resting electrocardiography (ECG) may be performed as part of the test prior to cycling. All tests will be supervised by the lead investigator who has appropriate first aid training, with access to medical personnel readily available at each participating hospital site. To reduce the risk of musculoskeletal injury from sudden or excessive exertion, functional movement screening will be conducted prior to testing, and a gradual ramping protocol will be used. Each test will include an appropriate warm-up and cool-down period.

There are no potential risks associated with EEG recordings; however sanitiser/disinfectant for cleaning skin where the cap is to be placed. Participant could potentially experience an allergic reaction, or sensitive skin following chemotherapy compared to normal. EEG hydro-links will be a fitted into the cap, therefore also maintaining a sterile field. Although the EEG system is not diagnostic, any incidental EEG findings which warrants further investigation will be reported to the direct care team. These would include any EEG findings that also suggest underlying symptoms. The EEG data would not be analysed in real time and does not have any direct diagnostic value, but as part of good clinical practice, as outlined above, will be discussed with them if any incidental findings are noted. Additionally, the cognitive function test battery and physical evaluation can potentially lead to fatigue, frustration, or emotional disturbance. The study will be conducted with close attention to patient safety and will not interfere with or alter the standard of care that patients would normally receive. If they do suffer any other symptoms or they become concerned in any way prior to their next study visit or after the study has finished, they should contact the lead investigator or any investigators named on this sheet (chief or principal investigators) prior to their next visit.

Home-based exercises carry potential risks in both supervised and unsupervised sessions. Such risks include adverse events: cardiovascular events, neurological complications such as neuropathy-related balance issues, as well as general safety such as falls and musculoskeletal injuries. All exercises (aerobic and resistance/strength) will include adaptations to suit the participants’ individual circumstances. For example, providing a chair to perform resistance band exercises. Family, friends, or caregivers must be present in the household for both supervised and unsupervised sessions. If the participant is unable to contact the emergency services, someone will be present to assist. Support will also be provided during supervised exercise sessions. The participant will be provided with an emergency contact list. Calls between the lead investigator (or member of the research team) will provide an opportunity to screen for any injuries and side effects of treatment (e.g. neuropathy) which will inform any appropriate adaptations to exercise sessions.

Regarding the use of multivitamins (Forceval), the principal investigator during the screening process will exclude participants with any contraindications to taking these multivitamins (e.g. impaired kidney and liver function, listed in Section 6.2) which can be indicated through markers and medical history on patient medical records. Routine blood count checks conducted as part of the standard of cancer care will provide additional monitoring for potential adverse changes. The multivitamin used will be dispensed and recorded by the participating hospital, ensuring that the direct care team (oncologists, oncology nurses, and PIs) is aware of supplementation but remains blinded to group assignment. Awareness of multivitamin use alongside blood count results may help the direct care team identify potential concerns and, in collaboration with the research team, determine whether further steps are needed. Participation may be terminated if clinically indicated to safeguard patient wellbeing.

Venepuncture for the collection of blood samples for neuroplasticity assessment carries minor risks, including bruising, haematoma, infection, and vasovagal syncope. To minimise these risks, all procedures will be carried out by a trained healthcare clinician or nurse using an aseptic technique. Pressure will be applied to the puncture site following sample collection to reduce the likelihood of bruising and haematoma. Participants will be observed post-procedure to monitor for any adverse reactions, including signs of fainting or discomfort, ensuring a proper and appropriate response if required. The blood samples for the brain markers are not for clinical use and will be analysed at the end of the testing period, i.e., when the last participant is tested at Visit 3. Thus, the findings of these samples will not be communicated to a clinician (member of the direct care team or general practitioner (GP)) as it will be weeks/months from when they were collected.

Before the first visit to the hospital for testing, the Informant Questionnaire On Cognitive Decline In The Elderly (IQCODE, self-report form) (via Qualtrics, <https://lancasteruni.eu.qualtrics.com>) and the Mini-Cog (WebCog, <https://rishik4.github.io/webcog/#/>) will be used to assess cognitive function, and physical activity engagement via the Physical Activity Readiness Questionnaire for Everyone (PAR-Q+) and International Physical Activity Questionnaire (IPAQ) can be found in the appendices. All questionnaires will be completed to check eligibility to take part, and if the participant is eligible, they will be scheduled into a testing slot at their participating hospital site (visit 1). It is important to highlight that these questionnaires cannot be used as clinical assessment tools in this setting and that the lead researcher is not qualified to provide a diagnosis of cognitive impairment. If the patient is concerned by the results of the questionnaires or would like further information, they will be advised to contact their direct care team or GP. A GP letter can be provided by the research team to the patient to give details about the screening questionnaires used and evidence about potential cognitive decline if they seek to speak to their GP for advice.

## 3. OBJECTIVES AND OUTCOME MEASURES/ENDPOINTS

### 3.1. Primary objective

1. We will use physiological blood-based markers of (VEGF and BDNF) to establish if prehabilitation promotes an improvement in brain health in colorectal cancer patients prior to starting chemotherapy.
2. We will use neuroscientific measures of brain function (EEG) and cognitive outcomes at baseline and post-chemotherapy to determine if prehabilitation neuroplasticity reduces the negative effects of chemotherapy on the brain.

### 3.2. Secondary objectives

1. We will measure changes in the FACT-COG questionnaire in colorectal cancer patients at Study Visit 1 and at 3 months after chemotherapy cessation to determine if prehabilitation improves quality of life.

### 3.3. Outcome measures/endpoints

We will use a range of physiological (CPET, blood panels), neuroscientific (EEG) and psychological (cognitive function test battery, quality of life questionnaire) to determine if our prehabilitation intervention improves chemo-brain and quality of life in patients with colorectal cancer. Our intervention will be delivered across Lancashire, with the aim of reducing health inequalities and negative health outcomes associated with cancer treatment in the region.

### 3.4. Primary endpoint/outcome

We will measure brain function at three-time points; baseline (after diagnosis/before prehabilitation, visit 1), after 3-4 weeks prehabilitation (before chemotherapy, visit 2) and after the final round of chemotherapy (3-4 days after infusion of the final round, visit 3). Outline in Figure 1. Chemotherapy typically range from to 2-8 rounds of infusion, with each round being spaced out by 2-3 weeks. The maximum duration of prehabilitation participation is approx. 28 weeks; however, the intervention may be extended (endpoint to visit 3) due to delays to treatment from chemotherapy-related side effects (e.g., low blood cell counts). Thus, time is needed to be given to participants in order to recover, reduce the risk of infection and worsened side effects. Participation in the prehabilitation programme will not influence or delay the timing of any scheduled chemotherapy treatment.

### 3.5. Secondary endpoint/outcome

There will be a 3 month follow up following chemotherapy to assess changes in quality of life. This data collection is feasible to complete within a 2 year period.

### 3.6. Table of endpoints/outcomes

| **Objectives** | **Outcome Measures** | **Timepoint(s) of evaluation of this outcome measure (if applicable)** |
| --- | --- | --- |
| **Primary Objective** | We will use physiological blood-based markers of (VEGF and BDNF) to establish if prehabilitation promotes an improvement in brain health in colorectal cancer patients prior to starting chemotherapy. | Visit 1 (day 0), Visit 2 (3-4 weeks), Visit 3 (8-24 weeks) |
|  | We will use neuroscientific measures of brain function (electroencephalography (EEG)) and cognitive outcomes at baseline and post-chemotherapy to determine if prehabilitation neuroplasticity reduces the negative effects of chemotherapy on the brain. | Visit 1 (day 0), Visit 2 (> 3 days before first chemotherapy), Visit 3 (3-4 days after final chemotherapy) |
| **Secondary Objectives** | We will measure changes in the FACT-COG questionnaire in colorectal cancer patients at Study Visit 1 and at 3 months after chemotherapy cessation to determine if prehabilitation improves cognitive-related quality of life. | Visit 1 (day 0) and Follow Up (3 months following chemotherapy) |

## 4. STUDY DESIGN

A multi-centre randomised control trial (RCT) using a prehabilitation group (exercise and nutrition programme) and a control group (standard care).

## 5. STUDY SETTING

Participants will be recruited via weekly clinician reviews by the principal investigators at the East Lancashire Teaching Hospitals Trust (Subar), Lancashire Teaching Hospitals NHS Trust (Williamson), and University Hospitals Morecambe Bay NHS Trust (Ton). Recruitment and testing will be conducted at each participating site. These Trusts are affiliated with Lancaster University.

**East Lancashire Teaching Hospitals Trust (ELTH):** The research environment at the East Lancashire Hospitals NHS Trust is dynamic and rapidly advancing, reflecting its commitment to delivering high-quality, patient-centred research. The East Lancashire Hospitals NHS Trust has expanded its research portfolio over the past decade, actively participating in national and international studies across specialities, including oncology. In collaboration with the NIHR Clinical Research Network Greater Manchester, East Lancashire Hospitals NHS Trust has enhanced its research infrastructure, providing patients with greater access to innovative clinical trials.

**Lancashire Teaching Hospital NHS Trust (LTHTR):** The research environment at Lancashire Teaching Hospital NHS Trust is nationally recognised, underpinned by its designation as an NIHR Clinical Research Facility in partnership with the Lancashire Care NHS Foundation Trust and Lancaster University. The Trust activity supports over 150 research projects across a broad spectrum of clinical areas, including oncology, consistently surpassing NIHR recruitment targets. The Lancashire Teaching Hospital NHS Trust is committed to research excellence is further demonstrated by its strategic partnership with Lancaster University, fostering a robust clinical academic faculty and supporting the development of early phase clinical trials.

**University Hospitals Morecambe Bay NHS Trust (UHMBT):** The research environment at University Hospitals Morecambe Bay NHS Trust is collaborative, reinforced by strong partnerships with Lancaster University and the University of Cumbria across its three main hospital sites: Furness General Hospital, Royal Lancaster Infirmary, and Westmorland General Hospital. These formalised agreements focus on joint initiatives in research, innovation, education, and workforce development; aiming to enhance health outcomes across the region. The University Hospitals Morecambe Bay NHS Trust actively participates in clinical research areas of oncology. The Trust research and development department comprises a multidisciplinary team that supports the governance, performance, and quality of research studies.

## 6. PARTICIPANT ELIGIBILITY CRITERIA

All eligible participants will be given a patient information sheet at the first meeting with their direct care team. This will be determined before the randomisation process. The eligibility criteria were carefully decided to ensure that participants were medically appropriate for selection. Participants will be considered for participation once they meet the inclusion criteria and none of the exclusion criteria, as detailed below.

### Inclusion criteria

*The inclusion criteria*: participants aged 60-85 years and diagnosis of stage II or III colorectal cancer undergoing chemotherapy, including fluorouracil, capecitabine, or oxaliplatin. Confirmation of patient medical records of participants being scheduled to receive either neoadjuvant or adjuvant chemotherapy, with suitability and tolerability to receive chemotherapy confirmed by their direct care team via patient medical records (e.g., dependent how the patient’s health and recovery following surgery). The duration between surgery and the start of chemotherapy is typically 6+ weeks, dependent on the patients’ recovery. Participant is fluent in English. Prehabilitation benefits those most who are not habitually active; therefore, exclusion criteria include structured exercise in the 6 months prior to the point of consent. Equal numbers of males and females will be included.

### 6.2. Exclusion criteria

*The exclusion criteria:* participants with co-morbidities which impact the metabolic response to exercise, for example diabetes, current musculoskeletal injury/physically rendering them unable to undergo CPET, atrial fibrillation, palliative disease, haematological malignancy, synchronous cancer disease, and lack the capacity to consent. Evidence of pre-existing cognitive impairment, including diagnosis of dementia, other neurodegenerative disorders, or clinically indicated mild cognitive impairment. Participant has no diagnosis of profound hearing loss. Participants without access to the internet within their home will not be able to take part due to the online element of the exercise programme.

*Further exclusion criteria related to multivitamins (Forceval):* Participants will be excluded if they have hypercalcaemia, haemochromatosis, or allergic to peanut or soya. Concomitant use of medications that interact with Forceval, such as phenytoin or tetracycline antibiotics will also lead to exclusion. Individuals with impaired kidney or liver function (e.g., chronic kidney disease) will not be eligible.

## 7. STUDY PROCEDURES

### 7.1*.* Recruitment

The principal investigator or suitably trained member of the direct care team with delegated duties to do so, at each participating hospital site will review lists of colorectal cancer patients weekly throughout the recruitment window. Potential participants meeting the inclusion criteria will be passed to the lead investigator who will select patients while consulting with the lead clinician of the project (Subar). Recruitment will continue for a period of 8-12 months. At three monthly periods the recruitment rate will be assessed to gauge progress and address recruitment issues.

#### 7.1.1. Screening

Initial eligibility will first be assessed by the principal investigator or a suitably trained and delegated direct care team member at each participating hospital site, who will screen for potential participants meeting the eligibility criteria through patient medical records through on-site system(s) (Section 6.1 and 6.2). Eligible patients must receive adjuvant chemotherapy (after surgery or radiotherapy) confirmed within their treatment plans to be suitable for study participation. This usually refers to the period of six or more weeks from the point at which the patient begins chemotherapy after surgery. If a suitable patient is identified, the principal investigator/member of the direct care team will liaise with the lead investigator to arrange a date and time for screening, coordinated around the patient’s oncology appointment. No identifiable patient information will be disclosed at this stage. The direct care team member will briefly introduce the study to the participant in the clinical consultation and obtain verbal consent from the lead investigator to approach them or join the consultation to discuss the study further. The lead investigator will enter the consultation and discuss the study. Patients who expresses interest will be given a study participant information sheet and provide verbal consent to be contacted in a one-week follow-up call. Those who confirm interest within this time period will provide verbal consent to proceed and will begin the formal screening process during the telephone call. Screening questionnaires will include the International Physical Activity Questionnaire (IPAQ) to confirm the absence of structured exercise participation within the previous six months, a key exclusion criterion. The physical activity readiness questionnaire (PAR-Q+) will be used to assess whether participants are safe to exercise with medical clearance, if needed. These questionnaires were completed during telephone calls. The IQCODE and Mini-Cog will be administered to screen for cognitive impairment. The IQCODE assesses self-reported cognitive changes in older adults, and the Mini-Cog self-reported cognitive function. These questionnaires will be sent to the potential participant via an online link following the telephone call which needs to be returned as soon as possible, before a testing visit can be scheduled (if eligible). Screening questionnaires (IPAQ, PAR-Q+, IQCODE, and Mini-Cog) will be completed before the baseline visit (visit 1) to confirm eligibility. Eligible participants will also be re-contacted, verbal consent to take part will be obtained, and the first research visit will be arranged.

After completing the above, if an individual is found to be ineligible during screening with the lead investigator, efforts will be made to minimise disappointment and reduce distress. They will be encouraged to seek advice about exercise chemotherapy from their direct care team. Additionally, the patient will be directed to the Macmillan Cancer Support to access the Physical Activity and Cancer booklet (<https://www.macmillan.org.uk>) as well as the exercise guidelines provided by Cancer Research UK (<https://www.cancerresearchuk.org>). If there are any concerns that the individual expresses from the cognitive function questionnaires (IQCODE or Mini-Cog) then the researcher will advise for them to speak to their direct care team or GP. The researcher can provide details of the questionnaires in a GP letter to provide support. The questionnaires are not diagnostic tools.

#### 7.1.2. Payment

Participants will be reimbursed for travel costs to and from each study visit. Car travel will be reimbursed at a rate of 45p per mile, which reflects with standard mileage reimbursement within the UK. Participants will complete the claims forms to receive payment through the project’s funding. Public transport costs will be reimbursed at the exact price paid for tickets or fares. Participants must provide proof of purchase such as a ticket or receipt to ensure transparency and accurate reimbursement across all modes of travel.

### 7.2. Consent

Verbal consent for the patient to be approached about this study will first be obtained by the principal investigator or direct care team member. The direct care team member will then introduce the lead investigator, who will inform the patient about the study. They patient will be provided with the participant information sheet and ask for verbal consent to follow-up one week later via telephone. At the follow-up telephone call, if the potential participant expresses interest in the study, they will undergo the screening for cognitive function and physical activity. The participant will then receive another call with the outcome of these assessments and, if eligible, arrangements will be made for the first study visit. At Visit 1 (baseline assessment), written informed consent will be obtained, signed, and dated only after the participant has received full information about the study and confirmed their decision. Participants remain free to withdraw from the study at any point without giving a reason and without prejudice.

### 7.3. The randomisation scheme

Using a permuted block randomiser, we will randomise 86 patients with colorectal cancer into Group 1: Intervention (Prehabilitation; n = 43) and Group 2: Control (Standard Care With No Prehabilitation; n = 43).

#### 7.3.1. Method of implementing the randomisation/allocation sequence

A computer-generated randomised sequence will be performed at the Lancaster University Health Statistics Department. Randomisation will be stratified by sex, staging, and rounds of chemotherapy required. This sequence will be delivered to the investigating team via sealed envelopes. Eligible participants will be randomised on a 1:1 basis to standard care: exercise and nutrition. To ensure 1:1 randomisation allocation, the lead investigator will review allocation at the end of the recruitment period. Allocation details will be contained in sealed envelopes. Eligible patients who have consented will be given a unique participant number. The participants will be randomised after the baseline CPET.

### 7.4. Baseline data

All participants recruited in both the intervention and control group will undergo a baseline measurement on cardiopulmonary fitness (CPET), blood samples (BDNF and VEGF), brain activity (EEG), cognitive function, and cognitive-related quality of life at visit 1. This baseline data will be collected before group assignment (assigned following CPET).

### 7.5. Study Intervention and Assessments

#### 7.5.1. Intervention (Prehabilitation)

**Exercise Programme**

Participants will be taught how to perform exercises safely at home. For the intervention group, the CPET-derived parameters at baseline such as VO_2_ peak will be used to prescribe an individualised exercise programme at the precise target moderate intensity for the aerobic components of the intervention (60% of VO_2_ peak). This will consider the participants’ capabilities to perform the exercises along with functional fitness assessments performed at the baseline visit. The exercise programme will be designed, delivered, and overseen by the lead investigator, who will hold a valid exercise-instructor level 4 qualification in cancer care. The supervised exercise programmes can be delivered by a suitably qualified exercise instructor in the lead investigator’s absence. The intervention is underpinned by the Theory of Planned Behaviour, which suggests that intention to exercise is shaped by attitudes, social norms, and perceived controls. To encourage adherence, the approach aims to promote positive beliefs about exercise, provide support from family/friends/peers to reinforce social norms, and enhance perceived control by helping participants integrate physical activity into their routine and environments, especially before starting chemotherapy.

The exercise programme will be performed four times per week before and during chemotherapy. There will be 2 supervision sessions and 2 unsupervised sessions, self-paced sessions that will be performed remotely from the participant’s home. An online supervised exercise sessions will be delivered by the lead investigator. The online sessions will be held on Microsoft Teams call, in which participants will be provided with links to join the call. The exercise programme will involve 40-minute sessions of aerobic and strengthening exercise: to include 5-minute warm up, 20-minute aerobic exercises at moderate intensity (60% V̇O_2_ peak determined by the CPET), 10-15 minutes of strength/resistance training, followed by a 5-minute cool down. The strengthening exercises will focus on upper and lower muscle groups. Different grades of resistance bands and dumbbells will be provided and used. Alternative and equivalent exercises, through regressions and progressions, will be employed by the lead investigator based on the participants mobility capabilities. For unsupervised sessions, participants will be provided with an exercise booklet, along with exercise videos created for the study, to be performed independently. Participants will be educated in the primary exercises of this programme with progression and regression versions of each exercise. The exercise programme will be updated on a frequent basis but will continuously include the primary exercises with slight variations or the inclusion of similar exercises to prevent participants losing interest in the exercise programme. The participants involvement in the exercise programme and any changes to their individual exercise prescription will be managed by the lead investigator in liaison with the clinical team, if required. For all supervised and unsupervised exercise sessions, participants in the prehabilitation group will be required to have a family member, carer, or friend present to provide immediate assistance in the event of an emergency incident. Participant will provide the contact details of their next of kin to the lead investigator prior to the supervised exercise sessions. A sample of the exercise programme can be found in the appendix.

**Psychological Support**

In addition to the supervised and unsupervised exercise sessions, participants will receive weekly telephone calls from a member of the research team. These calls will provide behavioural support to help participants achieve their weekly exercise targets and will be structured using the Theory of Planned Behaviour. Discussions will focus on identifying individual motivators and barriers to exercise, with this information used to adapt both the exercise sessions (through progressions and regressions) and the overall approach to supporting each participant’s engagement in the intervention. The lead investigator is not a healthcare professional. If any medical concerns are raised during the call, participants will be advised to contact their direct care team or the oncology helpline. If urgent situations they will be instructed to call 999 or 111.

**Nutrition**

At the first visit (baseline), participants will have a nutritional blood test using the Abridged Scored Patient-Generated Subjective Global Assessment (abPG-SGA)^5^ that will collect several markers: full blood count, urea and electrolytes, glucose, liver function tests, magnesium & phosphate, calcium & albumin, clotting function, copper, zinc, selenium, iron, ferritin, B12, folate, manganese, C-reactive protein and vitamin D). The lead investigator will be present at all initial visits and with the research team’s support will collect the blood samples. Samples will be labelled before being sent to the pathology laboratory at each participating site. Any queries regarding results from the nutritional blood tests will be assessed by the dietitian as part of standard of care.

Prehabilitation participants will be given a multivitamin (Forceval Capsules, United Kingdom), to be taken orally once per day throughout the duration of the intervention (before and during chemotherapy). The multivitamins will be taken in addition to the exercise programme. Compliance will be checked by asking participants to bring in used capsule trays at visits 2 and 3.

Forceval capsules are a multivitamin supplement that contains a combination of 24 essential vitamins (Vitamin A, D_2_, B_1,2,6,12_, C, E, Biotin, Nicotinamide, Pantothenic Acid and Folic Acid), minerals and trace elements (Calcium, Iron, Copper, Phosphorus, Magnesium, Potassium, Zinc, Iodine, Manganese, Selenium, Chromium, Molybdenum). Each vitamin and mineral plays a vital role in the efficient daily maintenance of the body. Multivitamin administration will be managed and dispensed as part of the standard of care and will be monitored by the participating hospital, with appropriate documentation and oversight. Patients with contraindications to multivitamin use will be excluded prior to enrolment (Section 6.2). Blood samples will be collected in this study and provided to the hospital for routine analysis of nutritional tests (abPG-SGA), in which the research team will collect nutritional marker data relevant to this study following analysis. As part of standard care, the direct care team will have access to the test results and will be able to take action, if required. Notably, only participants within the prehabilitation arm will receive multivitamin supplementation as an adjunct to standard care. Beyond the prescriber, the other members of the direct care team will remain blinded to group allocation to minimise bias. If the research team needs to report any concerns during the collection of blood samples, they will follow the notification cascade steps from the reporting workflow in Section 8.2.

**Support During Intervention**

To further support participants’ efforts to increase their activity and engage in exercise sessions, they will be provided with online resources, including exercise programmes and videos. Participants will also have the option to join a private Microsoft Teams group chat, moderated by the lead investigator. In this group chat:

1. Announcements about weekly dates/times of online supervised exercise sessions will be posted.
2. Participants can indicate which sessions they will attend.
3. All exercise programmes, exercise videos and additional resources will be archived in the group chat for easy access.
4. Links for online supervised exercise sessions will be posted in the chat.

When participants opt in (via the consent form), they are informed that names and email addresses may be visible to others during Microsoft Teams exercise sessions. All participants will be reminded to keep any visible information strictly confidential and to avoid using it for any purpose outside the study, including contacting other participants directly. The use of Microsoft Teams is covered by the University’s existing Office 365 data protection documentation, ensuring full compliance with institutional and legal requirements. This is further explained in the Data Protection Impact Assessment (DPIA). Members of the Microsoft Teams group will include the lead investigator and participants in the prehabilitation group.

If a participant wishes to opt out of the Microsoft Teams group, they will still receive all the same resources, updates, and Teams call links (for the supervised exercise sessions) via email.

**Habitual Activity**

All participants, including those in the standard care group, will have their habitual physical activity measured using the activity device (ActiGraph wGT3X-BT1, Ametris, Florida). This device is a triaxial accelerometer that captures bodily acceleration at a sampling rate of 30 Hz. Data will be processed using ActiLife software, with step counts derived using the Freedson algorithm and extracted in 10-second epochs. Participants will wear the device on the non-dominant wrist for seven consecutive days during three distinct periods: the first week of prehabilitation before chemotherapy, the first week of chemotherapy, and the week before the final chemotherapy cycle. The device will provide measures of total movement, moderate to vigorous physical activity (minutes), non-sedentary time (minutes), and step count (steps per day) over each 7-day period. Data from these measures will be transferred directly from the device when participants return to their participating hospital site. Habitual physical activity measures obtained from this device will be used to compare the prehabilitation and standard care groups.

The prehabilitation group will use the Xiaomi Smart Band 9 (Xiaomi, China) to motivate and monitor activity during the exercise programme (both supervised and unsupervised) and daily living activities. This wrist-worn device includes an accelerometer, gyroscope, six-axis motion sensor, heart rate sensor (photoplethysmography, PPG), and blood oxygen sensor. Participants will wear the device on their dominant hand throughout the intervention. It provides objective quantification of total daily activity through step count (steps per day), distance (metres), standing time (minutes), and time spent in moderate to high-intensity activity (minutes). Moderate intensity reflects activity that elevates heart rate to 50–75% of the individual’s estimated maximum, while high intensity exceeds 70% of maximum, making conversation difficult. Based on established thresholds for step count, fewer than 3,000 steps indicates low activity, and 3,000–10,000 steps indicates moderate activity.

Data collection will be synchronised with the fitness application, which can remotely export measures every two weeks as CSV files accessible only to the research team. The lead investigator will provide prompts during supervised sessions and check-in calls to encourage device use. If participants experience skin irritation from the silicone straps, these can be replaced with nylon straps. Any day containing null values will be treated as non-wear or device failure. The device will be collected at the final visit (visit 3). If a participant withdraws from the study, they will be asked to return the device to their participating hospital or, alternatively, will be provided with a pre-paid postage envelope.

**Adherence To The Intervention**

Adherence to the intervention will be assessed in four ways: (1) marking attendance at supervised sessions, (2) weekly check-ins during telephone calls, (3) null data values from the Xiaomi device, and (4) data from the activity device (ActiGraph) measuring minutes of moderate intensity or greater activity, excluding habitual activity outside prescribed sessions.

**Ongoing care**

Both groups will be seen in the outpatient setting at time intervals predetermined by their direct care team. Each participant’s cancer care plan will not be affected by study visits or the prehabilitation programme (intervention group). Visits will be arranged, where possible, to coincide with appointments with the direct care team to minimise the burden of travel.

#### 7.5.2. Assessments

In summary, cardiopulmonary fitness (CPET), blood sampling, brain activity, cognitive function, and cognitive-related quality of life will be assessed at visit 1 (baseline). Visits 2 and 3 will include repeated assessments of cardiorespiratory fitness, blood sampling, brain activity, and cognitive function. At the three-month follow-up after the final chemotherapy session, cognitive-related quality of life will be reassessed via telephone or post. Visit 1 (baseline) takes place 3–4 weeks before chemotherapy (time-to-treatment). Visit 2 occurs within 72 hours of starting chemotherapy and visit 3 will take place 3–4 days after the last chemotherapy session. See Figure 1.


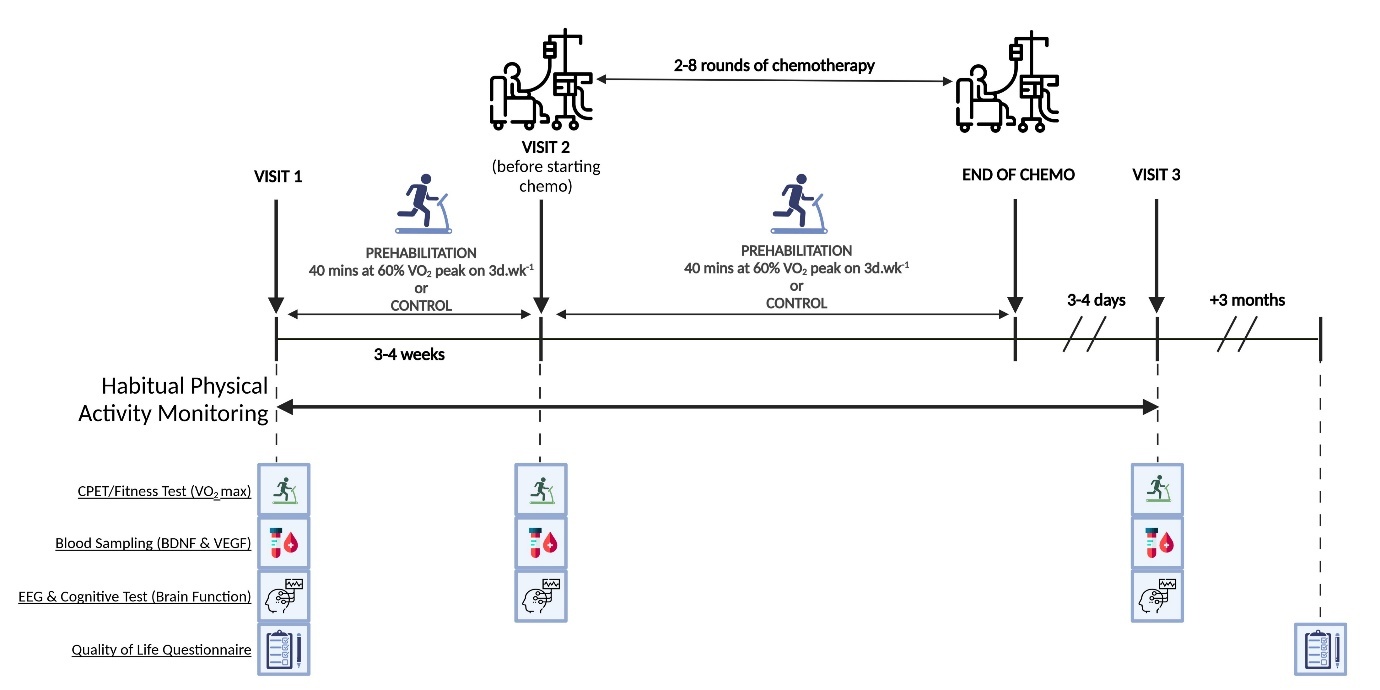


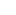

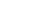

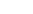

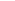


*Figure 1. Illustration of the timeline of the study visits, assessments, and intervention.*

**Cardiopulmonary Exercise Test (CPET) & Functional Fitness**

Participants will be advised to take their regular medication but to avoid caffeine, alcohol, cigarettes, and strenuous exercise on the day of each visit. For two hours prior to testing, patients must fast except for water. They should wear comfortable sports clothing and appropriate shoes. The CPET will be performed using a cycle ergometer at each participating site. Cycle ergometry allows accurate determination of work rate, enabling evaluation of the VO2–work rate relationship. Gaseous analysis will be conducted using a gas analyser, and continuous oxygen saturation will be monitored with a pulse oximeter.

After explaining the test protocol to the participant, a resting data collection period (rest phase/baseline) will be followed by resistance-free pedalling (unloaded cycling phase), then a continuous, gradual, uniform increase in work rate until the participant reaches their limit of tolerance (incremental phase). Pedalling frequency will be maintained between 60 and 80 rotations per minute. Increments will be adjusted based on the participant’s physical fitness level, aiming to reach maximal effort.

Additionally, overall functional fitness will be assessed prior to CPET to track changes in participants’ abilities across testing visits. This will allow personalised adjustments to the exercise programme during prehabilitation, based on any functional limitations. Following the Senior Fitness Test protocol^8^, participants will perform sit-to-stand tests, arm curls, grip strength using a dynamometer, sit and reach, back scratch, up-and-go test, six-minute walk test, and three-minute step test. Their scores will be compared to age- and sex-specific norms (men and women aged 60–64, 65–69, 70–74, 75–79, 80–84, and 85–89 years) outlined by Jones and Rikli^8^. Normal score ranges and test descriptions are provided in the appendix.

**Brain Activity and Cognitive Function**

The participant will be fitted with an EEG cap using saline-based hydro-links during each testing visit at the participating sites. The participant will then complete a resting measure, followed by a modified change detection task to assess memory while EEG activity is recorded.

Electrophysiological measures of brain activity will be used to assess brain function and are sensitive to neural changes resulting from chemotherapy-induced cognitive impairment. In chemotherapy-induced cognitive impairment, there is an overall slowing of brain activity. Using the EEG system, brain activity during the cognitive test battery will be recorded throughout each testing session. Subsequent analysis will focus on frequency band oscillations, including theta (4–8 Hertz) and alpha (8–13 Hertz) bands. Power spectral density will be calculated to quantify the relative contribution of each band, enabling identification of neural activation patterns associated with cognitive performance in subdomains such as executive function, attention, memory, and processing speed. Specific regions of interest will be defined to examine spatial variation in oscillatory activity; however, all hydro-link sites will be assessed.

The cognitive test battery will be objectively assessed using the National Institutes of Health (NIH) Toolbox Cognitive Domain (<http://nihtoolbox.org/>). This toolbox comprises a series of researcher-administered, computer-adaptive tests that take approximately 20 minutes to complete. The adaptive feature of the tests is designed to minimise practice effects, as well as floor and ceiling effects. We will examine age-standardised scores from four tests to assess all subdomains: executive function and attention will be assessed using the Flanker Inhibitory Control and Attention Test; memory will be assessed using the List Sorting Working Memory Test; and processing speed will be assessed using the Oral Symbol Digit Test.

To ensure that any deficits in auditory instructions and cues are not due to hearing loss, an audition test (Words-In-Noise Test) will be used to differentiate between hearing loss and chemotherapy-related cognitive impairment. The tests will be administered by the researcher, and participants will use a digital tablet with audio guidance for each test. Each test will take between three and seven minutes to complete.

The EEG and cognitive assessment will last approximately 50 minutes: 15 minutes for setup and 35 minutes to complete the resting measure and cognitive test battery, with breaks between tests. Detailed descriptions of these tests have been previously published, and the battery has been validated in adults and normed for individuals aged 3 to 85 years^9,10^.

**Blood Samples**

Chronic exercise can increase brain-derived neurotrophic factor (BDNF), which in turn has neurotrophic and neuroprotective properties that can improve cognitive function. BDNF and vascular endothelial growth factor (VEGF) will be measured using an Enzyme-Linked Immunosorbent Assay (ELISA) from blood serum. The lead investigator will be present at all initial visits, and a research nurse or suitably trained staff member will collect, process, and label the samples before storing them at each participating site. All samples will be transported via courier from each site to Lancaster University for ELISA analysis following the final participant test visit.

Blood will be drawn by site staff from a cubital vein into a S-Monovette 7.5 ml Z tube (with clotting activator; Sarstedt), left to coagulate for 30 minutes at room temperature, and centrifuged at 2000 g at room temperature for 10 minutes. The serum supernatant will be stored at -80°C in 0.5 ml aliquots within one hour of blood sampling. Blood samples will be used to determine BDNF and VEGF levels. The timing of blood samples will be consistent across testing visits at each site (± 2 hours).

**Cognitive-Related Quality of Life**

To evaluate cognitive-related quality of life, we will use the FACT-Cog questionnaire. This validated tool is currently used to measure cognitive-related quality of life in cancer patients and its impact on daily activities. The questionnaire includes several subscales: perceived cognitive impairment, perceived cognitive abilities, impact on quality of life, and comments from others. Each item is scored from 0 (not at all) to 4 (very much), with some items reverse scored.

We will measure changes in cognitive-related quality of life using the FACT-Cog questionnaire in colorectal cancer patients at Study Visit 1 and again at three months after chemotherapy cessation to determine whether prehabilitation improves quality of life post-treatment.

### 7.6. Withdrawal criteria

Any study participant can withdraw from the study at any time by contacting the lead investigator or any member of the research team, without providing a reason and without affecting their legal rights. If they withdraw, the information collected up to that point cannot be erased and may still be used in the project analysis.

### 7.7. Storage and analysis of clinical samples

Lancaster University does not hold a Human Tissue License. However, according to the Human Tissue Authority, blood serum is not classified as relevant material when extracted using standardised methods that guarantee the sample is completely acellular. This study will use a method that renders the material acellular. Therefore, there are no external storage regulations for serum. Nonetheless, Lancaster University maintains its own sample tracking database, and samples will be analysed and stored under the NHS Research Ethics Committee’s Favourable Opinion. The serum supernatant will be stored at -80°C in 0.5 ml aliquots within one hour of centrifugation following blood sampling. All samples will be transported by a licensed biological samples courier from each participating site to Lancaster University for analysis using an Enzyme-Linked Immunosorbent Assay (ELISA) on blood serum. No samples will be stored outside the listed institutions (Lancaster University and participating hospitals involved in this study). Samples will not be shared with other institutions, either within or outside the UK.

Following Lancaster University’s disposal procedures, blood samples will be handled as hazardous clinical waste, disposed of via clinical waste bins, and destroyed by incineration. All blood samples will be destroyed once analyses of blood-based markers of brain health via ELISAs are complete at the end of the study. All procedures will comply with the seventh revision of the Declaration of Helsinki and Good Clinical Practice guidelines. Blood samples will be stored in accordance with Human Tissue Authority Codes of Practice. The study protocol will be preregistered on the ISRCTN registry, and findings will be published within 12 months of trial completion. The ISRCTN registry will be updated throughout the study and with any outputs generated.

### 7.8. Definition of the end of Study

The final follow-up at three months, using the quality of life questionnaire (FACT-Cog) completed by the last participant, will signal the end of the study. However, not all participants will have identical cancer treatment plans, as the number of chemotherapies rounds and their duration may vary.

## 8. SAFETY REPORTING

### 8.1. Definitions

| **Term** | **Definition** |
| --- | --- |
| **Adverse Event (AE)** | Any untoward medical occurrence in a participant to whom an intervention has been administered, including occurrences which are not necessarily caused by or related to that intervention. |
| **Adverse Reaction (AR)** | An untoward and unintended response in a participant to the intervention |
| **Serious Adverse Event (SAE)** | A serious adverse event is any untoward medical occurrence that:   - results in death - is life-threatening - requires inpatient hospitalisation or prolongation of existing hospitalisation - results in persistent or significant disability/incapacity - consists of a congenital anomaly or birth defect   Other ‘important medical events’ may also be considered serious if they jeopardise the participant or require an intervention to prevent one of the above consequences.  NOTE: The term "life-threatening" in the definition of "serious" refers to an event in which the participant was at risk of death at the time of the event; it does not refer to an event which might have caused death if it were more severe. |
| **Serious Adverse Reaction (SAR)** | An adverse event that is both serious and, in the opinion of the reporting investigator, believed with reasonable probability to be due to one of the Study treatments, based on the information provided. |

###
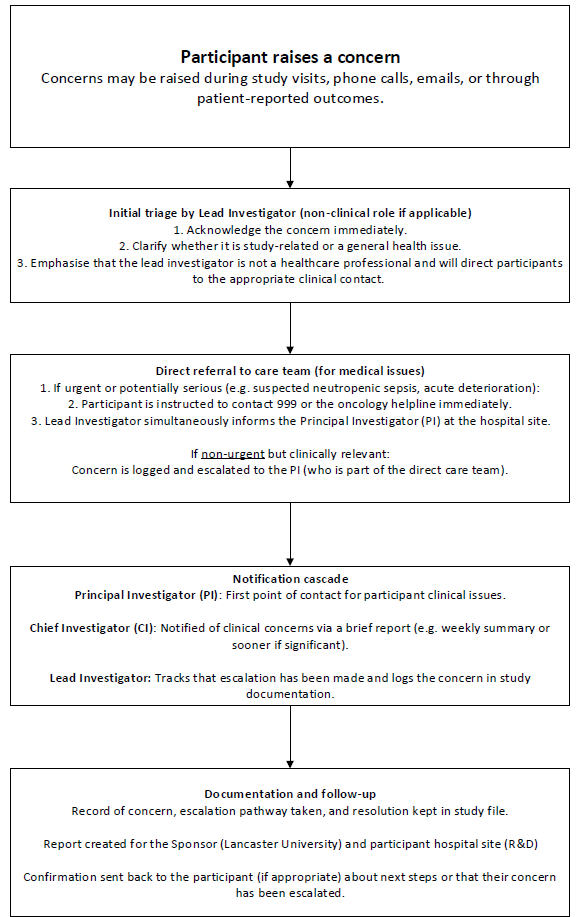
8.2. Workflow of reporting concerns to the direct care team and documentation

## 9. STATISTICS AND DATA ANALYSIS

### 9.1. Sample size calculation

Sample sizes were calculated in G*Power based on repeated measures mixed models to detect medium effect sizes (f = 0.25), with a power of 0.8 and an alpha of 0.01. A sample size of 34 participants per group meets the power criteria. To allow for 20% attrition, 43 participants per group will be recruited. While the power calculation is based on the primary outcome, the study is sufficiently powered for each of the secondary outcomes.

### 9.2. Planned recruitment rate

Total recruitment rate will be 86: 43 participants in the interventional group and 43 participants in the control group.

### 9.3. Statistical analysis plan

#### 9.3.1. Summary of baseline data and flow of patients

Baseline comparability between the randomised groups will be assessed descriptively, without statistical testing, in accordance with CONSORT guidelines for randomised trials. Variables used to assess baseline characteristics will include:

- Age (continuous)
- Sex (categorical: male/female)
- Cancer Stage (categorical: stage II and III)
- Chemotherapy regimen (categorical)
- Anthropometrics (continuous: e.g., height and weight)
- Comorbidity status (categorical)
- Education (categorical)
- IPAQ physical activity level and PAR-Q (categorical)
- Mini-Cog score (ordinal)

These variables will be summarised using appropriate descriptive statistics: means and standard deviations for continuous normally distributed data, medians, and interquartile ranges for non-normally distributed data, and counts and percentages for categorical variables. No inferential statistical comparisons will be conducted on baseline data, in line with best practice in RCT reporting.

The flow of participants through each stage of the trial (enrolment, allocation, follow-up, analysis) will be presented in a CONSORT flow diagram. This will include the number of participants assessed for eligibility, excluded (with reasons), randomised, receiving the allocated intervention, lost to follow-up, and included in the final analyses. Reasons for exclusion or withdrawal will be recorded and reported where available.

#### 9.3.2. Primary outcome analysis

Anonymised data spreadsheets will be password protected and transferred on encrypted storage devices to Lancaster University for analysis. All data will be analysed on Lancaster University computers and backed up to the Lancaster University servers.

We will employ mixed ANCOVAs with Group (control vs prehabilitation) as the between-subjects factor and Time (Study Visit 1, Study Visit 2) as the within-subjects factor, with physiological outcomes as the dependent variables. Staging will be included as a covariate. Significance level will be corrected to account for multiple testing. Additionally, we will employ mixed ANCOVAs with Group (control vs prehabilitation) as the between-subjects factor and Time (Study Visit 1, Study Visit 3) as the within-subjects factor. Cancer staging and rounds of chemotherapy will be used as covariates, with EEG spectral ratios/cognitive outcomes as the dependent variables. The threshold of significance will be corrected to account for multiple testing.

#### 9.3.3. Secondary outcome analysis

We will employ mixed ANCOVAs with Group (control vs prehabilitation) as the between-subjects factor and Time (Study Visit 1, 3-month follow-up after chemotherapy cessation) as the within-subjects factor, with quality-of-life score on the FACT-Cog as the dependent variable. Staging and rounds of chemotherapy will be included as covariates.

### 9.4. Procedure(s) to account for missing or spurious data

Efforts will be made throughout the study to minimise missing data. Strategies include flexible scheduling of study visits, where possible, and prompt data entry with certification against source documents. The reasons for any missing data will be systematically recorded, including participant withdrawal, clinical contraindications loss to follow-up, or technical issues.

## 10. DATA MANAGEMENT

### 10.1. Data collection tools and source document identification

Participants will be issued a unique participant identification number. All study encounters, including consent, will be recorded in the participants’ native hospital notes. Data will be entered into a purpose-built master database held on a secure NHS server. Members of the immediate research team (lead investigator and co-investigators) will be responsible for entering this data. Physical paper copies will also be filed in a Trial Master File and stored in a designated locked cabinet in the principal lead’s office at each participating site.

Some electronic data will be automatically generated and transcribed into the master spreadsheet. Laboratory analysis data will be stored on secure Lancaster University computer servers.

Questionnaires will be issued to participants at their baseline visit and requested to be completed. If participants are unable to complete them on the day, pre-paid envelopes will be provided for return. The second round of questionnaires will be issued at the three-month follow-up assessment (FACT-Cog) and participants will be asked to return them via pre-paid envelopes if they are unable to complete them over the telephone. A letter will accompany the questionnaires, indicating that completion is voluntary and there is no obligation should the questionnaire cause anxiety or distress. Contact details of the lead investigator will be provided for participants wishing to discuss any aspect of the questionnaire.

### 10.2. Data handling and record keeping

The research team will preserve the confidentiality of participants in accordance with the Data Protection Act. All data collected will be accurately recorded and securely stored by the team, and no identifiable information will be accessible to individuals outside the study team. Clinical, demographic, CPET, blood panel, and EEG data will be anonymised at the point of collection before being transferred outside the participating hospital’s clinical areas to Lancaster University. Data will be identified only by ID numbers, with personal information accessible only via a code held by the lead and chief investigators.

All data transfers will be conducted via the secure, encrypted university cloud (OneDrive) managed by Lancaster University and verified by the participating hospital site’s IT team. The only non-digital data will be the signed consent forms, which will be securely stored in the principal lead’s office at each participating site and transferred to the chief investigator’s office at Lancaster University once all visit assessments are complete. All collected data will reside on Lancaster University’s secure, encrypted network, accessible only through password-protected computers within the university. After the study concludes, data will be stored for the minimum period specified in the protocol and then destroyed accordingly.

Participants retain the right to withdraw from the study at any time without providing a reason. If a reason is given, it will be recorded as well as any ‘loss to follow-up.’ Participants will be asked to consent to the retention of any data collected up to the point of withdrawal.

### 10.3. Access to Data

There will be no access to identifiable participant data outside the research team at any stage of the project. Trust guidelines on data protection and General Data Protection Regulations (GDPR) guidance will be adhered.

## 11. ETHICAL AND REGULATORY CONSIDERATIONS

### 11.1*.* Research Ethics Committee (REC) review & reports

Before the initiation of the trial, all trial-related materials—including consent forms, the participant information sheet (PIS), protocol, and other relevant documentation—will be submitted to the relevant Research Ethics Committee (REC) for approval, as well as to the Health Research Authority via the Integrated Research Application System (IRAS).

Any subsequent amendments to these documents will be submitted for further approval. Participants’ rights to refuse participation in the trial without providing a reason must be respected. After a participant has entered the trial, the clinician remains free to provide alternative treatment to that specified in the protocol at any stage if they consider it to be in the participant’s best interest. The reasons for doing so must be documented.

Following randomisation, participants cannot change their allocated group (i.e., prehabilitation or standard care), and data will be analysed according to this allocation. However, participants remain free to withdraw from testing, the intervention (prehabilitation group), or follow-up at any time without giving a reason and without prejudice to their treatment.

### 11.2. Public and Patient Involvement (PPI)

The PPI steering committee includes four individuals with lived experience of colorectal cancer (including chemotherapy and “brain fog”-type symptoms) and two carers of people undergoing chemotherapy. Initial meetings have helped shape the study design, including the delivery of the intervention and testing visits. For example, the committee reviewed the frequency and duration of the proposed testing sessions to ensure they are realistic alongside treatment schedules and minimise participant burden. Reflections from these early discussions have directly informed considerations of participant burden, such as scheduling tests flexibly around hospital appointments (where possible), providing comprehensive instructions for home-based exercises (e.g., offering different formats such as programme booklets and videos), recommending multivitamin capsules once a day rather than a drink version, and addressing barriers and facilitators to exercising at home.

The PPI steering committee has reviewed participant-related documents (e.g., participant information sheet and consent form) to ensure they are accessible, sensitive, and avoid overwhelming language. The committee will continue to meet throughout the study and advise on strategies to support participants who may experience chemotherapy-related symptoms. Current recommendations include offering rest breaks, splitting sessions into shorter segments (e.g., aerobic and resistance exercises separated during the day instead of back-to-back), and providing prompts for study activities.

All meetings will be chaired by the lead investigator to ensure effective time management and that every voice is heard. Members will receive INVOLVE rates of payment, and travel expenses will be reimbursed. A support plan will be implemented to encourage engagement and minimise burden for steering group members. At the end of the study, members will receive the final report and will be invited to contribute to dissemination plans and outputs to ensure findings are communicated in a way that is meaningful to patients and carers. All patient-facing documents will be reviewed by the steering group for any submissions or amendments to NHS ethics.

### 11.3. Regulatory Compliance

The trial will be conducted in compliance with the approved protocol, the Declaration of Helsinki (2013), the principals of Good Clinical Practice (2016), the UK Data Protection Act, and the UK Policy Framework for Health and Social Care Research.

### 11.4. Protocol compliance

The trial steering committee (TSC) will be established by and operate under the authority of Lancaster University [Sponsor]. Membership will comprise the lead investigator, chief investigator, co-investigators, three independent academics external to the study team, and a PPI Advisor drawn from the PPI Steering Committee. Mrs Becky Gordon, as the Sponsor’s representative, will also attend TSC meetings to maintain direct oversight.

The TSC is authorised to provide independent oversight of the study’s conduct, progress, and compliance with protocol, ethics, and regulatory requirements. It will advise the lead investigator and research team on operational issues and emerging challenges, such as risks to participant safety, data integrity, and overall study delivery, and recommend mitigation actions as needed. The TSC will report concerns or escalate decisions to the Sponsor or Funder [North West Cancer Research] when necessary to ensure proper governance is maintained.

An authorised individual, external to the project and not employed by Lancaster University, will be responsible for data monitoring. This includes overseeing data quality, participant safety, and overall trial integrity by reviewing data for accuracy, completeness, and compliance with protocols. In the event of concerns, this individual will conduct unbiased interim analyses, review safety data, identify potential risks or benefits, and make recommendations for protocol modifications or, if necessary, trial termination to ensure participant safety and the scientific validity of the study. The lead and chief investigators, along with the lead clinician, will support this authorised individual in monitoring activities as required.

### 11.5. Data protection and patient confidentiality

The study will be conducted in compliance with the approved protocol, the Declaration of Helsinki (2013), and the UK Data Protection Act. The principal investigator, or a suitably trained member of the direct care team with delegated duties, will review lists of colorectal cancer patients (via medical records) weekly throughout the recruitment period. Patients will be given the PIS at their initial outpatient clinic appointment. Verbal consent will be obtained to contact potential participants by phone. The lead investigator of the research team will follow up via telephone and, if the prospective participant agrees to take part, obtain informed consent at the participant’s baseline assessment visit. The lead investigator will code the samples; no identifying information will be available to anyone outside the research team. All documentation will be accurately recorded and secured on password-protected databases and in a physically held site file.

Participants retain the right to withdraw from the study at any time without providing a reason. If a reason is given, it will be recorded along with any ‘loss to follow-up.’ Participants will be asked to consent to the retention of any data collected up to the point of withdrawal.

Blood samples will be transported to Lancaster University for data analysis and will not be shared externally outside the research team for additional research. All data will be stored only on Lancaster University computers, and loaned laptops will be used to access data, with access restricted to authorised research members involved in this study.

### 11.6. Financial and other competing interests for the Chief investigator, Principal Investigators at each site and committee members for the overall study management

On behalf of all investigators, we declare that we have developed the research questions and authored the research proposal herein. We further have no conflicts of interest to declare relating to this work.

### 11.7. Amendments

If an amendment is needed during a study, the Amendment Tool must be completed to determine its category (A, B or C), which reflects its impact on participating sites; separate from whether the amendment is substantial or non-substantial. The Sponsor, Lancaster University, is responsible for authorising the amendment and completing the declaration section of the Amendment Tool; no amendment should be submitted without prior sponsor authorisation.

Once submitted, the amendment and relevant documents must be shared with affected participating sites. Category A and B amendments require to be reviewed within 35 days, assess whether they can support and implement the changes. Category C amendments still need to be shared to take appropriate administrative action, but they do not require a capacity and capability review.

Communication to affected participating sites is important to agree implementations plans, discuss any impact on activities or costs, and address concerns. If a site cannot support the amendment, options such as site closure should be discussed. Investigators and participating sites should work to the most recent approved documents relevant to their role in the study.

### 11.8. Post Study Care

The chief investigator will have control of the data and act as the custodian for all data generated by the study and will be responsible for ensuring the secure storage of this data for a period of 12-36 months following the end of the study; this includes data from CPET, EEG, cognitive assessments, and quality of life measures.

### 11.9. Access To The Final Study Dataset

Only the lead and chief investigators and the research team will have access to the code which will be linked to patient identifiable details kept in the Trial Master File.

## 12. DISSEMINIATION POLICY

### 12.1. Dissemination policy

This work is expected to yield several publications in reputable international journals, and the findings will be presented at regional and international conferences. Following study completion, we will host a Results Open Evening for participants, GPs, scientists, and the general public. If the intervention is successful, we will have identified a potential target group of patients likely to benefit from prehabilitation and will seek to implement our intervention at scale.

### 12.2. Authorship eligibility guidelines and any intended use of professional writers

*Authorship on the overall study report will be granted to the Lead Investigator (first author), Chief Investigator (senior/final author), and Co-Investigators (co-authors) based on substantial contributions to the study’s design, data acquisition, analysis or interpretation, and manuscript preparation. No professional medical writers will be involved, or if they are, they will be appropriately acknowledged for their contributions.*

## **13. REFERENCES**

1. El-Sayes, J., Harasym, D., Turco, C. V, Locke, M. B. & Nelson, A. J. Exercise-Induced Neuroplasticity: A Mechanistic Model and Prospects for Promoting Plasticity. The Neuroscientist 25, 65–85 (2018).
2. Franklyn, J. et al. Geographical variations in long term colorectal cancer outcomes in England: a contemporary population analysis revealing the north–south divide in colorectal cancer survival. Surg Endosc 37, 5340–5350 (2023).
3. Huang, G. H., Ismail, H., Murnane, A., Kim, P. & Riedel, B. Structured exercise program prior to major cancer surgery improves cardiopulmonary fitness: a retrospective cohort study. Supportive Care in Cancer 24, 2277–2285 (2016).
4. Lambert, J. E., Hayes, L. D., Keegan, T. J., Subar, D. A. & Gaffney, C. J. The Impact of Prehabilitation on Patient Outcomes in Hepatobiliary, Colorectal, and Upper Gastrointestinal Cancer Surgery: A PRISMA-Accordant Meta-analysis. Ann Surg 274, 70–77 (2021).
5. Lambert, J., Subar, D. & Gaffney, C. Prehabilitation for gastrointestinal cancer surgery. in Recent strategies in high risk surgery (ed. Faintuch, J.) vol. In Press (Springer Nature, 2024)
6. North West Cancer Research. North West Regional Report. (2021).
7. Rich, B., Scadeng, M., Yamaguchi, M., Wagner, P. D. & Breen, E. C. Skeletal myofiber vascular endothelial growth factor is required for the exercise training-induced increase in dentate gyrus neuronal precursor cells. J Physiol 595, 5931– 5943 (2017).
8. Jones, C.J. and Rikli, R.E., 2002. Measuring functional. The Journal on active aging, 1, pp. 24-30.
9. Weintraub, S., Dikmen, S.S., Heaton, R.K., Tulsky, D.S., Zelazo, P.D., Bauer, P.J., Carlozzi, N.E., Slotkin, J., Blitz, D., Wallner-Allen, K., and Fox, N.A., 2013. Cognition assessment using the NIH Toolbox. Neurology, 80(11_supplement_3), pp.S54-S64.
10. Weintraub, S., Dikmen, S.S., Heaton, R.K., Tulsky, D.S., Zelazo, P.D., Slotkin, J., Carlozzi, N.E., Bauer, P.J., Wallner-Allen, K., Fox, N. and Havlik, R., 2014. The cognition battery of the NIH toolbox for assessment of neurological and behavioral function: validation in an adult sample. Journal of the International Neuropsychological Society, 20(6), pp.567-578.

##

## 14. APPENDICES

**Appendix –Schedule of Assessment**

| **Procedures** | **Visits (3 visits & follow up)** | | | | |
| --- | --- | --- | --- | --- | --- |
|  | **Screening** | **Baseline** | **Assessments** | | **Follow Up** |
|  |  | **Visit 1** | **Visit 2** | **Visit 3** |  |
| Eligibility Assessment | / |  |  |  |  |
| Informed Consent | / | / |  |  |  |
| Screening Questionnaires | / |  |  |  |  |
| Demographics | / | / |  |  |  |
| Medical History | / |  |  |  |  |
| Physical Examination |  | / |  |  |  |
| Randomisation |  | / |  |  |  |
| Functional Fitness |  | / | / | / |  |
| Cardiopulmonary Exercise Test |  | / | / | / |  |
| Randomisation |  | / |  |  |  |
| Electroencephalogram |  | / | / | / |  |
| Cognitive Test Battery |  | / | / | / |  |
| Blood Samples |  | / | / | / |  |
| Quality Of Life Questionnaire |  | / |  |  | / |
| Nutrition Assessment |  | / | / | / |  |
| Physical Activity Monitor |  | / | / | / |  |

**Appendix – Amendment History**

| **Amendment No.** | **Protocol version no.** | **Date issued** | **Author(s) of changes** | **Details of changes made** |
| --- | --- | --- | --- | --- |
|  |  |  |  |  |

**Appendix –Sample Exercise Programme**

| **Warm Up** | **Time (seconds)** | **Notes (All body weight)** |
| --- | --- | --- |
| Shoulder Rolls | 30 | **Regression**: Seated / **Progression**: Lightly March |
| Shoulder Shrugs | 30 | **Regression**: Seated / **Progression**: Lightly March |
| Hip Rotation | 30 | **Regression**: Seated / **Progression**: Larger Rotations |
| High Knees | 30 | **Regression**: Chair Or Wall Support Or Seated / **Progression**: Lightly March |
| Squat | 30 | **Regression**: Seated Squat / **Progression**: Add Weight |

| **Aerobic Exercise** | **Time (seconds)** | **Reps** | **Sets** | **Note** |
| --- | --- | --- | --- | --- |
| Arm Circles | 20-30 | 10 | 2-3 | Body Weight  **Regression**: Seated / **Progression:** Lightly March |
| Side Moving Jacks | 20-30 | 10 | 2-3 | Body Weight  **Regression**: No Arm Movement / **Progression**: Jumping Jacks |
| Marching (Front) | 20-30 | 10 | 2-3 | Body Weight  **Regression**: Seated / **Progression**: Weights |
| Shadow Boxing | 20-30 | 10 | 2-3 | Body Weight / Weights  **Regression**: Forward Pointing / **Progression**: Weights (Increase) |
| V Steps | 20-30 | 10 | 2-3 | Body Weight  **Regression**: Step Straight Forward / **Progression**: Punching Forward |

| **Resistance Exercise** | **Time (seconds)** | **Reps** | **Sets** | **Note** |
| --- | --- | --- | --- | --- |
| Front Raises | 20-30 | 10 | 2-3 | Resistance Band/Weight  **Regression**: Less tension/weight / **Progression**: Increase tension/weight |
| Side Raises | 20-30 | 10 | 2-3 | Resistance Band/Weight  **Regression**: Less tension/weight / **Progression**: Increase tension/weight |
| Seated Squats | 20-30 | 10 | 2-3 | Resistance Band/Weight  **Regression**: Less tension/weight / **Progression**: Increase tension/weight |
| Seated Abduction Extension | 20-30 | 10 | 2-3 | Resistance Band  **Regression**: Less tension / **Progression**: Increase tension |
| Seated Leg Press | 20-30 | 10 | 2-3 | Resistance Band  Regression: Less tension / Progression: Increase tension |

| **Cool Down** | **Time (seconds)** | **Repetitions** | **Sets** | **Notes (All body weight)** |
| --- | --- | --- | --- | --- |
| Overhead Stretch | 10 | 10 | 2 | Chair or Standing |
| Neck Stretch | 10 | 10 | 2 | Chair or Standing |
| Side Lean Stretch | 10 | 10 | 2 | Chair or Standing |
| Hamstring Stretch | 10 | 10 | 2 | Chair or Standing |
| Ankle Stretch | 10 | 10 | 2 | Chair or Standing |
